# Automatic segmentation of brain tumors in magnetic resonance imaging

**DOI:** 10.31744/einstein_journal/2020AO4948

**Published:** 2020-02-27

**Authors:** Layse Ribeiro Mascarenhas, Audenor dos Santos Ribeiro, Rodrigo Pereira Ramos

**Affiliations:** 1 Universidade Federal do Vale do São Francisco PetrolinaPE Brazil Universidade Federal do Vale do São Francisco , Petrolina , PE , Brazil .

**Keywords:** Diagnostic imaging, Brain neoplasms, Image processing, computer-assisted, Magnetic resonance imaging, Computer simulation

## Abstract

**Objective:**

To develop a computational algorithm applied to magnetic resonance imaging for automatic segmentation of brain tumors.

**Methods:**

A total of 130 magnetic resonance images were used in the T1c, T2 and FSPRG T1C sequences and in the axial, sagittal and coronal planes of patients with brain cancer. The algorithms employed contrast correction, histogram normalization and binarization techniques to disconnect adjacent structures from the brain and enhance the region of interest. Automatic segmentation was performed through detection by coordinates and arithmetic mean of the area. Morphological operators were used to eliminate undesirable elements and reconstruct the shape and texture of the tumor. The results were compared with manual segmentations by two radiologists to determine the efficacy of the algorithms implemented.

**Results:**

The correlated correspondence between the segmentation obtained and the gold standard was 89.23%.

**Conclusion:**

It is possible to locate and define the tumor region automatically with no the need for user interaction, based on two innovative methods to detect brain extreme sites and exclude non-tumor tissues on magnetic resonance images.

## INTRODUCTION

One of the most advanced and widely used techniques for viewing brain tumors is magnetic resonance imaging (MRI), ^( 1 )^ due to its ability to differentiate between different types of tissues, and because it is a non-invasive method of high anatomical resolution, which makes diagnosis less traumatic to patients. Thus, MRI has been an important tool for detection, surveillance and early diagnosis of tumors. ^( 2 - 4 )^

Although MRI is highly detailed, a diagnosis based only on human intelligence is cumbersome and time consuming, and subject to interpersonal variability, loss of information and eye fatigue. Also, during acquisition, images may show low quality, poor contrast, and the presence of artifacts, which pose difficulties to the human eye. ^( 5 )^ Moreover, the segmentation of tumor borders is usually a visual task and a manual procedure, which can only detect marked changes, ^( 6 )^ and is subject to great variability when interpreted by different radiologists and long processing times to achieve detailed segmentation. ^( 7 , 8 )^

In this light, artificial intelligence-based image processing systems, known as CAD (computer-aided diagnosis) have been developed to detect and/or evaluate abnormalities on imaging examinations, and help physicians in precision diagnosis and neurosurgery. ^( 9 )^ Image processing and analysis are used to develop the analytical skills of physicians ^( 10 )^ and shorten the time required for accurate evaluation, therapy planning and tumor surveillance. ^( 3 , 11 )^ These techniques provide radiologists with a second opinion to help understand medical images, improving diagnostic precision. ^( 12 )^

Although CAD systems have shown relative progress in their performance, there are still several issues to be overcome before its sensitivity can be improved. Also, automatic analysis of medical images has image-related issues that need to be overcome, such as noise that can affect pixel intensity and non-consistent image intensity. ^( 13 )^ Hence, a thorough investigation of alternative algorithms for all steps of a CAD system becomes relevant, from pre-processing to the final classification stage, using methods that can minimize MRI processing.

In this study, we developed a new algorithm for automatic segmentation of brain tumors on T1c, T2 and FSPGR T1c MRI sequences, using mathematical methods, as well as logical and morphological operations. After the analysis, the algorithm was able to segment the tumor region in agreement with the previous manual segmentation by radiologists (the gold standard).

## OBJECTIVE

To develop and validate a set of computational tools for automatic segmentation of brain tumors on magnetic resonance images.

## METHODS

The methodology was structured with a system consisting of 3 steps: pre-processing, segmentation and post-processing. This system operates with three different types of MRI: T1c, T2 and FSPRG T1c, in axial, coronal and sagittal views. Thus, all steps of the system were designed to operate with these three MRI modalities and any of the views. The system and its steps are described in table 1 .

**Table 1:** System steps and respective processes

Step 1 Pre-processing	Step 2 Segmentation	Step 3 Post-processing
Histogram equalization	MDC	Dilation
Intensity adjustment	Erosion	Hole filling
Binarization	MDA	Spatial convolution

MDC: detection by coordinate; MDA: detection by area.

This study was initiated after approval by the Institutional Review Board of *Universidade Federal do Vale do São Francisco* (UNIVASF), CAAE: 90399118.5.0000.5196 and opinion number 2.954.791.

### Research setting

This study was conducted in the city of Juazeiro (BA), in the Electrical Engineering Laboratory at the UNIVASF campus.

### Database

For this study, we reviewed 14 MRI images of brain tumors in real patients, confirmed by manual segmentation by specialist physicians, *i.e.* the gold standard, as marked on the images and described in the chart of each anonymous patients.

A database was built with 116 images provided by a radiology center in the city of Petrolina on an anonymous basis (private database). The machine used for examinations was the 1.5 T Intera by Philips Medical Systems, the slices were 3.5mm thick and the spacing between each slice was 3.85mm. Also, we used 14 images from the REpository of Molecular BRAin Neoplasia DaTa (public database), provided free-of-cost by The Cancer Imaging Archive (TCIA). Thus, the total sample consisted of 130 images (slices) from 14 patients of both sexes and different ages, of which 19 were T1c, 48 T2 and 63 FSPGR T1c sequences.

The images were in the Digital Imaging and Communications in Medicine (DICOM ^®^ ) format, with different resolutions for each type of sequence, namely: 256×256 (T2), 512×512 (FSPGR T1c) and 704×704 (T1c). Algorithms were studied and implemented in the MATrix LABoratory ^®^ (MATLAB ^®^ ) system, which consists of a platform using high-level programming language and an environment for algorithm development, data analysis and visualization, and numerical computing.

### Inclusion and exclusion criteria

We selected images previously diagnosed with hyperintense brain tumors in T1c, T2 and FSPGR T1c weightings and different planes (axial, sagittal and coronal), in patients of both sexes and different ages. The same number of images without tumors were included. Images with no previous diagnosis and with high noise density (extremely blurred) preventing visualization of brain structures were excluded from our cases.

### Pre-processing

Pre-processing consists of applying techniques to reduce artifacts and enhance images, for improving their quality and highlighting a region of interest (ROI) for more detailed visualization and better segmentation precision. ^( 14 , 15 )^ This step was based on image contrast enhancement to highlight the tumor region. All images have grey-level pixel intensities which were normalized between zero and 1.

### Histogram equalization

Differently from other imaging techniques, in MRI, the pixel intensity has no fixed value in respect to the tissue image, *i.e* ., the same tissue could have different intensities, which makes it difficult to adopt intensity features as sources of information when segmenting images. ^( 16 )^ The problem of non-standardized intensities is minimized by the process of normalization. Histogram equalization is applied to leave the desired areas brighter than the rest of the image, thus facilitating their extraction. ^( 2 , 17 - 19 )^ The histeq function ^( 20 )^ is used to obtain said result.

### Intensity adjustment

For intensity adjustment we used the imhmax and imhmin functions, ^( 20 )^ which suppress all maximums with grey values lower than a threshold *h* or all minimums higher than *h* . ^( 21 , 22 )^ These thresholds were pre-defined according to the occurrence of grey levels in the images (histogram), and two optimum intervals were defined for the set of images studied, based on the observation. Each MRI image varies with intensities closer to zero or 1, some being predominantly dark and others brighter. Therefore, according to the fragment area of imaging histograms, it was possible to determine the thresholds for intensity adjustment.

### Binarization

To separate the background and the ROI, we applied binarization aided by the imregionalmax function, ^( 20 )^ which identifies the pixels of greater intensity and returns a binary image whose maximum pixels are assigned a value of 1, and all others are assigned as zero. ^( 15 )^ After this process, the image has a smaller number of subregions, mostly disconnected, resulting in less data and shorter processing times in the following steps.

### Segmentation

The segmentation step separates the brain from adjacent areas, such as the meninges, skull bones and healthy tissues. ^( 16 )^ One of the innovations in this study is the algorithm for automatic segmentation of brain tumors, consisting of two methods: detection by coordinates and detection by area.

### Detection by coordinates

The first differentiator of this study is the detection-by-coordinates method (MDC), which automatically removes undesirable areas external to the brain, such as the skull bone, the meninges and subcutaneous fat, using only basic mathematical concepts and logical operations. The algorithm automatically marks four points around the brain known as top, bottom, left and right edges, and eliminates all regions located before the left-top edge and after the right-bottom edge. For this end, we considered that the head of the patient is in the central region of the image, which is the usual setting of an MRI, and the brain is delimited by pixels with intensities greater than zero.

Considering an image with dimensions X and Y, the coordinates of the left-top edge are determined by fixing the ordinate in the middle of the image (Y/2). For the top and bottom edges, the abscissa is fixed (X/2). The algorithm scans the row corresponding to Y/2 from left to right, and the column corresponding to X/2 from top to bottom, until it finds the first pixel greater than zero and creates two vectors with all pixels different than zero. The first element of each vector corresponds to the pixel of the left-top edge of the brain, and the last element corresponds to the opposite, *i.e.* , the right-bottom edge.

However, in most images, the skull bone has pixels with intensities greater than zero. The edges are marked in this region. Since the purpose is to define the brain, and the brain is at a small distance from the skull, which is virtually always the same from the anatomical standpoint, each edge is displaced by a value corresponding to such a distance, and fixed on the outer boundaries of the brain.

In addition, the algorithm also takes into consideration each type of view, since the brain will have different shapes in each situation. In the coronal and sagittal views, the images also contain the neck region, with pixels greater than zero. To avoid errors when marking the bottom edge, for instance, we estimate the distance between the lower part of the neck and the brain, displacing the bottom edge ordinate upwards, at a larger proportion.

**Figure 1:**
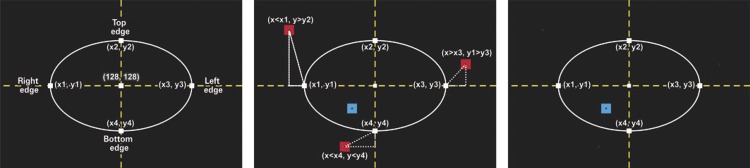
Segmentation using detection by coordinates. On the left, the edge points of the brain are marked. The second image illustrates the location of the external and internal subregions of the brain. Finally, all subregions external to the brain are excluded

After defining the brain by its edge points, the algorithm looks for subregions (labels) with coordinates lower than those of the left-top edge, and higher than those of the right-bottom edge. The labels are bounded by a rectangle using the bounding *box* parameter of the regionprops function. ^( 20 )^ The rectangle intercepts each label with at least two of its vertices. Thus, if at least one vertex of the bounding box containing the label is outside the limits of the brain edges, this indicates that the region is external to the brain and, therefore, a value of zero is assigned to all pixels of the corresponding label. This eliminates a great part of the skull, fat and meninges. Figure 1 illustrates the procedure for eliminating regions external to the brain using MDC.

### Detection by area

After applying detection by coordinates, the algorithm maintains the subregions inside the brain, which do not necessarily correspond to the tumor to be segmented. However, the tumor area resulting from detection by coordinates is, often times, larger than the arithmetic mean of the areas of all spurious elements that still remain. Thus, the second differentiator of this algorithm is the use of a two-dimensional parameter, *i.e.* , the area, to exclude non-tumor tissue inside the brain.

Before executing detection by area (MDA), the imerode function ^( 20 )^ was used to execute the erosion operation, which disconnects spurious elements and reduces their areas, forming new subregions with smaller areas. The MDA calculates the area of each resulting label and their arithmetic mean, assigning as zero the pixels of elements smaller than the arithmetic mean of the areas of all labels. This eliminates a great part or all of the brain tissue, leaving only the tumor.

**Figure 2:**
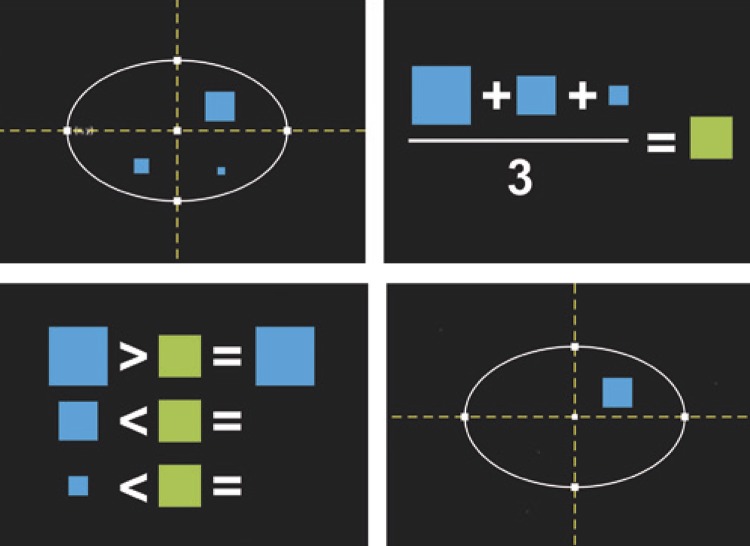
Eliminating subregions through detection by area. In the first column, the subregions internal to the brain are detected. In the second column, the arithmetic mean of the subregion areas is calculated. In the third column, the size of each subregion is compared with the arithmetic mean. In the last column, all subregions smaller than the mean are eliminated

Figure 2 illustrates the MDA, where the largest square in blue represents the tumor, other blue squares represent non-tumor tissue, and the green square represents the label corresponding to the arithmetic mean of the areas. Using logical operations (comparisons), the algorithm verifies the regions smaller than the mean area and excludes them, assigning a logical value of zero to their pixels. By doing so, ideally, one can eliminate as many undesirable elements as possible, and only the tumor remains.

### Post-processing

Morphological operators of dilation (imdilate) ^( 20 )^ were used to recover the tissues lost in the erosion process. To retain a larger region of the tumor, the binary mask is obtained by filling the holes in the dilated image (imfill). ^( 20 )^ Finally, the binary mask is applied to the original image by spatial convolution, to obtain a size close to the real size of the segmented elements, and then extract texture attributes. ^( 15 , 23 )^

### Evaluation of results

Quantitative results of the automatic segmentation were calculated by comparison between the gold standard and the proposed segmentation method. The metrics commonly used in the literature ^( 24 - 26 )^ to assess performance are the hit rate (HR), calculated by the number of true positive (TP) of the method compared with the gold standard; and the matching rate (MR), based on the number of false positive results (FP). In the cited studies, the HR and MR are respectively defined as:

$$\mathrm{HR}=\frac{\mathrm{TP}}{\mathrm{gold}\;\mathrm{standard}}\times 100\%$$

$$\mathrm{MR}=\frac{\mathrm{TP}\;–\;0.5\;\times \;\mathrm{FP}}{\mathrm{gold}\;\mathrm{standard}}$$

Higher HR values indicate a higher number of true positive pixels in the segmented area. The MR value determines how distant from the gold standard (GS) the segmentation is. The ideal MR value is 1, which means a perfect match between the gold standard and the proposed segmentation. Matching rate values close to 1 suggest that the proposed segmentation has a much higher rate of true positive than false positive results. ^( 16 )^

## RESULTS

**Figure 3:**
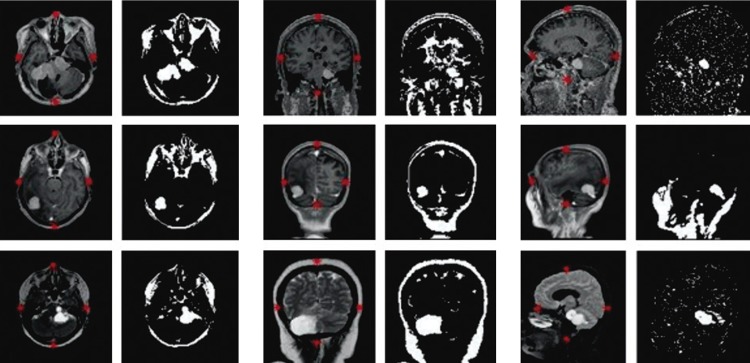
Results of pre-processing steps for different patients from the private database

The pre-processing step was implemented following the previous description. The images obtained at the end were treated with these pre-processing techniques. Figure 3 shows the output of the final process, after using histogram equalization, intensity adjustment for contrast correction, and binarization. The images on the right are binarized (they only show pixels with intensity zero or 1). Here we present the three types of MRI sequences and the three types of views of the brains of different patients.

The first, third and sixth columns show the images before processing, whereas the other columns show the images after pre-processing. The first row shows contrasted T1-weighted images, the second row shows T2-weighted images and the third row shows fast spoiled gradient echo T1-weighted images.

The segmentation step began with defining the brain by edge points and extraction of the regions external to them, followed by application of a morphological function of dilation, and finishing off with application of the MDA algorithm. Due to the erosion technique, which implies loss of pixels in certain regions, and the binarization process that converts the grey-scale image into only two levels of intensity, we used the morphological operations of dilation, hole filling and reconstruction, so that segmented regions could be rebuilt and returned to their original texture. The morphological procedure was used in these binary images to refine the margin and content of tumor images.

**Figure 4:**
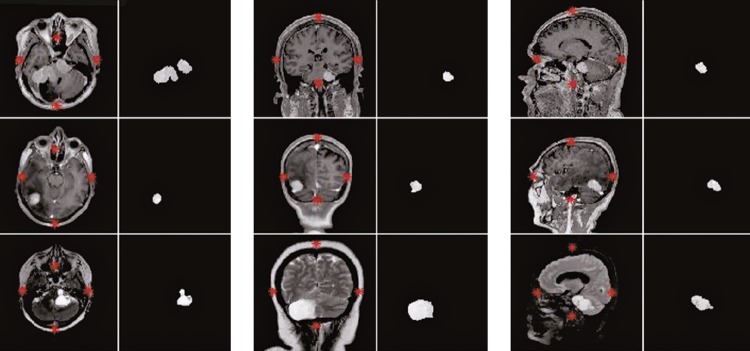
Final result of automatic segmentation in T1-, T2- and spoiled gradient echo contrasted T1-weighted magnetic resonance imaging

Figure 4 shows some final results after totally successful segmentation of MRI images of different patients, using T1c, T2 and FSPGR T1c sequences in the axial, coronal and sagittal planes. The first row shows segmentation of contrasted T1-weighted images of three different patients. The second row shows segmentation results for T2-weighted images. The third row shows segmentation results for fast spoiled gradient echo T1-weighted images.

### Comparative performance analysis of automatic segmentation

The MRI images used in the test had been manually segmented by radiologists. These results were considered as gold standard and used to efficiently assess the system. Table 2 shows TP, FP, FN and HR compared with the gold standard, as well as the respective MR values. In similar studies, authors obtained a HR of 86% and MR of 0.79, which was considered satisfactory. ^( 24 - 26 )^ In this study, the HR was 89.23% and the MR was 0.70, *i.e* ., quantitatively similar to those of previous authors, which indicates good accuracy of the proposed segmentation method. The best HR results were obtained for T2 and FSPGR-T1c sequences.

**Table 2:** Performance of segmentation by sequence type

Sequence	GS	TP	FP	FN	HR %	MR
T1c	33	27	11	6	81.81	0.65
T2	48	44	19	3	91.66	0.72
FSPGR T1c	49	45	21	4	91.83	0.70
Total	130	116	51	13	89.23	0.70

GS: gold standard; TP: true positives; FP: false positive; FN: false negative; HT: hit rate; MR: matching rate.

Matching rate results were satisfactory despite the significant number of FP. However, these FP were not more than the TPs, as seen in Alegro et al. ^( 23 )^ An innovative, robust image segmentation approach for extraction of brain tumors on MRI was developed by applying the Kernel function to the support vector machine (K-SVM), with HR and MR equivalent to 87.8% and 0.85, respectively. ^( 27 )^ To better compare the performance of the proposed segmentation algorithm and those found in the literature, the qualitative results of similar studies are presented in table 3 .

**Table 3:** Performance of segmentation algorithms used in the literature

Authors	Methodology	Performance assessment	
		HR (%)	MR
Alegro Mde C, Amaro Junior E, Lopes Rde D. Computerized brain tumor segmentation in magnetic resonance imaging. einstein (Sao Paulo). 2012;10(2):158-63. ^(23)^	SVM	94.00	-0.04
Deng W, Luo L, Lin X, Fang T, Liu D, Dan G, et al. Head and neck cancer tumor segmentation using support vector machine in dynamic contrast-enhanced MRI. Contrast Media Mol Imaging. 2017;2017:8612519. ^(24)^	SVM	86.00	0.89
Singh R, Agarwal P, Bhattacharya M. MR brain tumor detection employing Laplacian Eigen maps and kernel support vector machine. In: 2016 IEEE International Conference On Bioinformatics And Biomedicine (BIBM). IEEE. 2016;827-30. ^(27)^	FCM	72.80	0.43
Hsieh TM, Liu YM, Liao CC, Xiao F, Chiang IJ, Wong JM. Automatic segmentation of meningioma from non-contrasted brain MRI integrating fuzzy clustering and region growing. BMC Medical Informatics and Decision Making. 2011;11:54. ^(28)^	Kernel + SVM (K-SVM)	87.80	0.85
Gao F, Lin T. Application of Computer-Aided Diagnosis Technology in Brain Tumour Detection. NeuroQuantology. 2018;16(5):725-33. ^(29)^	Machine learning	97.17	-

HR: hit rate; MR: matching rate; SVM: support vector machine; FCM: fuzzy-c-mean algorithm; K-SVM: Kernel-based support vector machine.

## DISCUSSION

Pre-processing techniques could successfully eliminate a great portion of the brain and some regions of the encephalon, however some spurious elements remained such as the skull bone, lateral ventricles and the corpus callosum, which, in some instances, correspond to pixels with intensities close to or higher than those of tumor tissue and, therefore, are difficult to exclude. Also, we tested using the median filter, a tool commonly used for filtering noise in brain MRI. ^( 17 , 30 )^ However, results were not superior to those of the proposed method, and the processing time of the algorithm was a downside. In fact, the convolution operation with masks requires great computational effort. ^( 31 )^

The color of tumor tissue on T1-weighted images was very close to that of the brain. This can render the pre-processing step very difficult, since the criteria and thresholds used to enhance the tumor region end up being applied to other regions of the brain which are not of interest. This can generate healthy tissues connected to the tumor, even after using morphological operations, as well as increase the number of false-positive results after segmentation. Pre-processing results were better on T2-weighted and FSPRG T1c images, due to their better contrast between the hyperintense region and the rest of the brain.

When looking at figures 3 and 4, one can see that pre-processing and segmentation based on the previously described techniques showed good results with the use of a simple algorithm using basic features and logical operations. Table 4 shows the method used by some authors to improve the quality of MRI images of brain tumors and strip the skull, with results qualitatively comparable to those of this study.

**Table 4:** Comparative analysis of the pre-processing methodology proposed and those present in the literature

Authors	Methodology	Results
Isa IS, Sulaiman SN, Mustapha M, Karim NK. Automatic contrast enhancement of brain MR images using Average Intensity Replacement based on Adaptive Histogram Equalization (AIR-AHE). Biocybernetics and Biomedical Engineering. 2017;37(1):24-34. ^(15)^	Development of the AIR-AHE algorithm for automatic contrast enhancement of FLAIR MRI using the imadjust and stretchlim functions	The method showed good results along with other histogram equalization algorithms
Isselmou AE, Zhang S, Xu G. A novel approach for brain tumor detection using MRI Images. J Biomedical Sci Eng. 2016;9(10):44-52. ^(17)^	Use of the high-pass median filter and histogram equalization to improve image quality	The method improved image quality and provided excellent tumor segmentation results
Sujan M, Alam N, Noman SA, Islam MJ. A Segmentation based Automated System for Brain Tumor Detection. IJCA. 2016;153(10):41-9. ^(32)^	Brain extraction on FLAIR images using MATLAB ^®^ morphological operations, such as binarization, erosion, dilation and structuring elements	The method contributed to better image enhancement results, and greater accuracy when compared with other algorithms in the literature
Roy S, Maji P. A simple skull stripping algorithm for brain MRI. In: Eighth International Conference On Advances In Pattern Recognition (ICAPR) [Internet]. Kolkata: (IN); 2015 [cited 2019 Aug 20]. Available from: https://ieeexplore.ieee.org/abstract/document/7050671/ ^(33)^	Development of a method for skull stripping (S3) on T1-weighted images based on brain anatomy and image intensity	The performance of the algorithm is compared to BET and BSE, with satisfactory results
khandelwal P, Kaur G. Comparative study of different image enhancement technique. IJECT. 2016;7(2):116-21. ^(34)^	Comparison of contrast enhancement techniques (subtraction, contrast adjustment, erosion, gamma correction, inversion and thresholding)	The erosion technique yielded the greatest results
Kaur R, Chawla M, Khiva NK, Ansari MD. Comparative Analysis of Contrast Enhancement Techniques for Medical Images. Pertanika J Sci Technol. 2018;26(3):965-78. ^(35)^	Comparison of contrast enhancement techniques (neighborhood operation, median filter, imadjust and sigmoid function)	The sigmoid function and the neighborhood operation provided the greatest results
Shattuck DW, Sandor-Leahy SR, Schaper KA, Rottenberg DA, Leahy RM. Magnetic resonance image tissue classification using a partial volume model. Neuroimage. 2001;13(5):856-76. ^(36)^	Development of a method for skull stripping (BSE) on T1-weighted images using a border detector and a series of morphological operations	A robust method, which contributes for GM and WM segmentation of the brain, respectively
Smith SM. Fast robust automated brain extraction. Hum Brain Mapp. 2002;17(3):143-55. Review. ^(37)^	Development of a method for skull stripping (BET) on T1-weighted images	A robust, precise method applied to a range of MRI sequences
Roura E, Oliver A, Cabezas M, Vilanova JC, Rovira A, Ramió-Torrentà L, et al. MARGA: multispectral adaptive region growing algorithm for brain extraction on axial MRI. Comput Methods Programs Biomed. 2014 Feb 22;113(2):655-73. ^(38)^	Development of a method for skull stripping (MARGA) on axial views, based on the growth of the region	The MARGA had superior results when compared with the BET and BSE approaches
Somasundaram K, Mercina JH, Magesh Kalaiselvi ST. Brain Portion Extraction Scheme using Region Growing and Morphological Operation from MRI of Human Head Scans. IJCSE. 2018;6(4):298-302. ^(39)^	Development of a method for skull stripping based on growth of region and morphological operations (erosion, dilation and filling)	The results of the method are superior to those of existing methods (BET and BSE)
Kalavathi P, Prasath VB. Methods on Skull Stripping of MRI Head Scan Images—a Review. J Digit Imaging. 2016;29(3):365-79. Review. ^(40)^	Development of a method for brain extraction on T1-weighted MRI images based on median filter and morphological operations	The method provided results comparable to those of the BET and BSE methods and showed that the median filter did not improve segmentation

AIR-AHE: Average Intensity Replacement based on Adaptive Histogram Equalization; MRI: magnetic resonance imaging; FLAIR: fluid attenuation inversion recovery; MATLAB ^®^ : MATrix LABoratory ^®^ ; BET: brain extraction tool; BSE: brain surface extractor; GM: gray matter; WM: white matter; MARGA: multispectral adaptive region growing algorithm.

The MDC algorithm can mark the extremities of the brain on any view (axial, coronal or sagittal) of the brain. This improves accuracy, since the geometrical shape of the brain varies in the three different views. It also works when the head is located asymmetrically in the image, either horizontally or vertically, which is a common occurrence. ^( 30 , 41 )^ In addition, it offers different possibilities for extraction of regions outside the brain, and is applicable to images with hyperintense signal on different MRI weightings, since it does not depend on qualitative features, such as entropy and texture. Quite the opposite, it is based on the location of the brain inside the image, its anatomy and the intensity of surrounding pixels, similarly to the proposition of the S3 method. ^( 33 )^

The MDC showed satisfactory results regarding its functionality and scope, and it can be qualitatively compared with the algorithms presented in table 4 , with good performance in skull stripping. In cases where the area outside the brain is not completely eliminated, the morphological operation of dilation can subdivide said “remainders” into smaller portions, which can be eliminated using MDA. Thus, MDA works as a complement to segmentation by detection of coordinates. It is directly linked with the number of FP in post-segmentation, since the more non-tumor tissue can be eliminated, the fewer FP and the better the segmentation.

However, the MDA is flawed in situations where there are post-processed regions larger than the tumor, since there is a chance the tumor be smaller than the mean area of the existing subregions and eliminated as a consequence, which would decrease the number of TP, or produce images with tumor and non-tumor, increasing the FP. This can happen in views where normal tissues have similar pixel intensities to those of tumors, and the differentiation of these regions is made difficult by the pre-processing techniques adopted. In addition to these limitations, MDC and MDA were developed to segment only MRI images with hyperintense tumors.

The T1c sequence had lower HR and MR compared with other MRI weightings, due to images in which the pixel intensity of the brain region was similar to that of the tumor. Therefore, this led to a high number of connected FP which, in some cases, had a much larger area than the tumor; in these cases, the tumor was excluded and the segmented image had no tumor tissue, resulting in FN.

The failed cases using T2-weighting were due to the presence of ventricles in some coronal slices, as well as axial slices of the eyes and nose, with very high pixel intensity (close to 1), leading to a high number of FP, in addition to the tumor not being detected in three FN slices. This issue also led to failure of some axial FSPGR T1c images, which explains the high number of FP in this category.

Despite the significant number of FP, the HR and MC results were satisfactory, with a mean hit rate of 89.23% between the automatically segmented areas and the gold standard, pointing to good accuracy of the proposed segmentation method.

## CONCLUSION

The results obtained showed the proposed system was able to locate and define the tumor region without any user interaction, using an innovative method for automatic segmentation, simple and easy to implement.
