# Supplementary material for: Automatic segmentation of brain tumors in magnetic resonance imaging
Source: Einstein (Sao Paulo). 2020 Feb 27;18:eAO4948. doi: 10.31744/einstein_journal/2020AO4948 (PMC7053828; doi:10.31744/einstein_journal/2020AO4948)
Supplement: Translation

# Translation: Segmentação automática de tumores cerebrais em imagens de ressonância magnética

**Authors:** Layse Ribeiro Mascarenhas, Audenor dos Santos Ribeiro, Rodrigo Pereira Ramos

**Affiliations:** 1 Brasil Universidade Federal do Vale do São Francisco, Petrolina, PE, Brasil .

**Keywords:** Diagnóstico por imagem, Neoplasias encefálicas, Processamento de imagem assistida por computador, Imagem por ressonância magnética, Simulação por computador

## Abstract

**Objetivo:**

Desenvolver um algoritmo computacional aplicado a imagens de ressonância magnética, para segmentação automática de tumores cerebrais.

**Métodos:**

Foram utilizadas 130 imagens de ressonância magnética nas sequências T1c, T2 e FSPRG T1c e nos planos axial, sagital e coronal de pacientes acometidos com câncer cerebral. Os algoritmos empregaram técnicas de correção de contraste, normalização de histograma e binarização, para desconectar estruturas adjacentes do cérebro e realçar a região de interesse. A segmentação automática foi realizada por meio da detecção por coordenadas e por média aritmética da área. Operadores morfológicos foram utilizados para eliminar elementos indesejáveis e reconstruir a forma e a textura do tumor. Os resultados foram comparados com as segmentações manuais de dois médicos radiologistas, para determinar a eficácia dos algoritmos implementados.

**Resultados:**

Os acertos foram de 89,23% na correspondência entre a segmentação obtida e o padrão-ouro.

**Conclusão:**

É possível localizar e delimitar a região tumoral de forma automática, sem necessidade de interação com o usuário baseado em dois métodos inovadores de detecção dos extremos do cérebro e de exclusão dos tecidos não tumorais em imagens de ressonância magnética.

## INTRODUÇÃO

Uma das técnicas mais avançadas e difundidas para visualização de tumores cerebrais é a imagem de ressonância magnética (RM),^( 1 )^devido à sua capacidade de distinção entre diversos tipos de tecidos, e por ser um método não invasivo e de alta resolução anatômica, o que torna a obtenção do diagnóstico menos traumática para o paciente. Assim, a RM tem sido ferramenta importante para a detecção, o monitoramento e o diagnóstico precoces do tumor.^( 2 - 4 )^

Embora as RM apresentem alto grau de detalhamento, o diagnóstico feito somente com a inteligência humana é cansativo e demanda tempo, podendo ser suscetível às variações interpessoais, à perda de informação e à fadiga visual. Além disso, durante a aquisição, a imagem pode apresentar baixa qualidade, contraste ruim e presença de artefatos, que são problemas difíceis de lidar por meio do olho humano.^( 5 )^Ademais, o processo de segmentar as bordas da massa tumoral é normalmente realizado visualmente com a segmentação manual do tumor, que pode detectar apenas mudanças grandes,^( 6 )^obter grande variação na interpretação por diferentes radiologistas e demandar tempo para realização de segmentação detalhada.^( 7 , 8 )^

Diante disso, têm sido desenvolvidos sistemas computacionais de processamento de imagens e inteligência artificial, denominados diagnóstico assistido por computador (CAD - *computer-aided diagnosis* ), para detectar e/ou avaliar anormalidades em imagens médicas, e auxiliar os médicos na precisão do diagnóstico e em neurocirurgias.^( 9 )^A análise e o processamento da imagem são usados para desenvolver a competência analítica dos médicos^( 10 )^e reduzir o tempo necessário para a avaliação exata, o planejamento da terapia e o rastreamento da evolução do tumor.^( 3 , 11 )^Essas técnicas fornecem uma segunda opinião aos radiologistas, na compreensão de imagens médicas, melhorando a precisão do diagnóstico.^( 12 )^

Embora os sistemas CAD tenham alcançado relativo progresso em seu desempenho, ainda há vários problemas que precisam ser solucionados para melhorar sua sensibilidade. Além disso, a análise automática de imagens médicas apresenta problemas relacionados à imagem, que precisam ser superados, como ruídos que podem alterar a intensidade do *pixel* e a não uniformidade de intensidade da imagem.^( 13 )^Assim, a investigação completa de algoritmos alternativos para todas as etapas de um sistema CAD torna-se relevante, no que concerne desde a etapa de pré-processamento até a fase final de classificação, utilizando métodos que possam minimizar o processamento de RM.

Neste trabalho, foi desenvolvido um novo algoritmo de segmentação automática de tumores cerebrais em RM nas sequências T1c, T2 e FSPGR T1c, empregando-se métodos matemáticos, operações lógicas e morfológicas. Ao final da análise, o algoritmo foi capaz de segmentar a região tumoral em concordância com a segmentação manual dos radiologistas (o padrão-ouro).

## OBJETIVO

Desenvolver e validar um conjunto de ferramentas computacionais para segmentação automática de tumores cerebrais em imagens de ressonância magnética.

## MÉTODOS

A metodologia foi estruturada com um sistema composto por três etapas: pré-processamento, segmentação e pós-processamento. Esse sistema funciona com três tipos diferentes de RM: T1c, T2 e FSPRG T1c, nos cortes axial, coronal e sagital. Assim, todas as etapas do sistema foram projetadas para funcionar com essas três modalidades de RM e para qualquer um dos cortes. O sistema e as etapas são descritos na tabela 1 .

**Tabela 1:** Etapas e respectivos processos do sistema

Etapa 1 Pré-processamento	Etapa 2 Segmentação	Etapa 3 Pós-processamento
Equalização de histograma	MDC	Dilatação
Ajuste de intensidade	Erosão	Preenchimento de buracos
Binarização	MDA	Convolução espacial

MDC: método de detecção por coordenada; MDA: método de detecção por área.

A pesquisa foi iniciada após a aprovação do Comitê de Ética em Pesquisa da Universidade Federal do Vale do São Francisco (UNIVASF), CAAE: 90399118.5.0000.5196 e parecer 2.954.791.

### Local de realização da pesquisa

A pesquisa foi desenvolvida na cidade de Juazeiro (BA), no Laboratório de Pesquisa em Engenharia Elétrica, no campus da UNIVASF.

### Base de dados

Para o presente estudo, foram analisados 14 casos reais de tumores cerebrais de RM, cuja presença de tumores foi confirmada por meio da segmentação manual feita por médicos especialistas, o padrão-ouro, demarcada nas imagens e descritas no prontuário de cada paciente anonimamente.

Foi construída uma base de dados com 116 imagens cedidas por uma clínica médica de radiologia da cidade de Petrolina por anonimidade (base privada). A máquina utilizada para realização dos exames foi a Intera de 1,5 T da Philips Medical Systems, os *slices* (fatias) possuíam 3,5mm de espessura, e o espaçamento entre cada corte era de 3,85mm. Além disso, utilizaram-se 14 imagens obtidas da base de dados da *REpository of Molecular BRAin Neoplasia DaTa* (base pública) fornecida gratuitamente pelo *The Cancer Imaging Archive* (TCIA). Assim, totalizam-se 130 imagens (fatias) de 14 pacientes de ambos os sexos e idade variada, sendo 19 T1c, 48 T2 e 63 FSPGR T1c.

As imagens encontravam-se no formato de arquivos *Digital Imaging and Communications in Medicine* (DICOM^®^), apresentando diferentes resoluções para cada tipo de sequência, a saber: 256×256 (T2), 512×512 (FSPGR T1c) e 704×704 (T1c). Os algoritmos foram estudados e implementados no sistema *MATrix LABoratory* ^®^(MATLAB^®^), que consiste em uma plataforma que utiliza linguagem de programação de alto nível e ambiente de desenvolvimento de algoritmos, análise e visualização de dados e computação numérica.

### Critérios de inclusão e exclusão

Foram selecionadas imagens pré-diagnosticadas com tumores cerebrais em hipersinal nas ponderações T1c, T2 e FSPGR T1c e em diferentes cortes (axial, sagital e coronal) em pacientes de ambos os sexos e de idade variada. A mesma quantidade de imagens com ausência de tumor foi incluída. Imagens sem diagnósticos prévios e imagens com alta densidade de ruídos (extremamente borradas) que impediam qualquer visualização das estruturas do cérebro foram excluídas dos casos.

### Pré-processamento

O pré-processamento de imagens consiste na aplicação de técnicas para redução de artefatos e realce de imagens, que visam melhorar sua qualidade e destacar uma região de interesse (ROI), fornecendo sua visualização mais detalhada para melhorar a precisão de segmentação.^( 14 , 15 )^Esta etapa baseou-se no aprimoramento do contraste da imagem com o objetivo de evidenciar a região do tumor. Todas as imagens apresentam valores de intensidade de *pixel* em níveis de cinza e estas intensidades dos *pixels* foram normalizadas entre zero e 1.

### Equalização de histograma

Diferente de outras técnicas de imagem, na RM, a intensidade do *pixel* não tem valor fixado em relação à imagem do tecido, ou seja, o mesmo tecido pode ter intensidades diferentes, o que torna mais difícil a adoção de características de intensidade como fontes de informação na segmentação das imagens.^( 16 )^Essa falta de padronização de intensidade é reduzida por meio da aplicação de um procedimento de normalização. A equalização do histograma é aplicada para deixar áreas desejadas mais claras do que o resto da imagem e, assim, facilitar sua extração.^( 2 , 17 - 19 )^Utiliza-se a função *histeq* ^( 20 )^para obter tal resultado.

### Ajuste de intensidade

Para realizar o ajuste de intensidade, utilizaram-se as funções *imhmax* e *imhmin* ,^( 20 )^que suprimem todos os máximos com valor de cinza inferior a um limiar *h* ou todos os mínimos maiores que h.^( 21 , 22 )^Esses limiares foram prefixados de acordo com a ocorrência de níveis de cinza das imagens (histograma), sendo definidos empiricamente, a partir da observação, dois intervalos ótimos para o conjunto das imagens estudadas. Cada imagem de RM apresenta quantidade variável com intensidades mais próximas de zero ou de 1, sendo algumas predominantemente escuras e outras mais claras. Assim, de acordo a área de fragmentos do histograma das imagens, foi possível determinar tais limiares para o ajuste de intensidade.

### Binarização

Para separar o fundo da imagem e as ROI, aplicou-se a binarização, com auxílio da função *imregionalmax* ,^( 20 )^que identifica os *pixels* de maior intensidade e retorna uma imagem binária cujos *pixels* máximos adquirem valor 1, enquanto que, aos demais, são atribuídos valor zero.^( 15 )^Após esse processo, a imagem compõe-se de um número menor de subregiões, desconectadas em grande parte, implicando em menor quantidade de dados e menor tempo para o processamento nas próximas etapas.

### Segmentação

A etapa de segmentação do cérebro é responsável por separar o encéfalo das áreas adjacentes, como as meninges, os ossos do crânio e os tecidos saudáveis.^( 16 )^Uma das inovações deste trabalho é o algoritmo para realizar a segmentação automática de tumores cerebrais, que consiste em dois métodos: o de detecção por coordenada e o de detecção por área.

### Método de detecção por coordenada

O primeiro diferencial deste trabalho é o método de detecção por coordenada (MDC), que elimina, de forma totalmente automática, as regiões indesejáveis externas ao cérebro, como o osso do crânio, as meninges e a gordura subcutânea, utilizando apenas conceitos matemáticos básicos e operações lógicas. O algoritmo marca automaticamente quatro pontos ao redor do cérebro denominados de extremos superior, inferior, esquerdo e direito e elimina todas as regiões que se localizam antes do extremo esquerdo-superior e após o extremo direito-inferior. Para isso, considerou-se que a cabeça do paciente ocupa a região central da imagem, visto que as RM se apresentam dessa maneira, e observou-se que o encéfalo é delimitado por *pixels* com intensidade superior a zero.

Considerando-se uma imagem com dimensões X e Y, as coordenadas do extremo esquerdo-direito são determinadas fixando-se a ordenada na metade da imagem (Y/2). Para os extremos superior e inferior, é fixada a abscissa (X/2). O algoritmo varre a linha que corresponde a Y/2 da esquerda para a direita e a coluna correspondente a X/2 de cima para baixo, até encontrar o primeiro *pixel* maior que zero, criando dois vetores com todos os *pixels* diferentes de zero. O primeiro elemento de cada vetor corresponde ao *pixel* do extremo esquerdo-superior do encéfalo, e o último elemento equivale aos limites opostos, isto é, extremo direito-inferior.

No entanto, na maioria das imagens, o osso do crânio possui *pixels* de intensidades superiores a zero. Assim, os extremos são marcados nessa região. Como o interesse é delimitar o cérebro, e este encontra-se a uma pequena distância do crânio, que é praticamente invariável do ponto de vista anatômico, cada extremo é deslocado de um valor correspondente a tal distância, para serem fixados nos limites do cérebro.

Além disso, o algoritmo também leva em consideração cada tipo de corte, visto que o cérebro apresenta formas diferentes para cada situação. Nos cortes coronal e sagital, as imagens apresentam também a região do pescoço, a qual contém *pixels* maiores que zero. Para evitar erros na marcação do extremo inferior, por exemplo, estimou-se a distância entre a parte mais inferior do pescoço e do cérebro, deslocando-se a ordenada do extremo inferior para cima, em uma proporção maior.

Após delimitado o cérebro por seus pontos extremos, o algoritmo procura subregiões ( *labels* ) que possuam coordenadas menores que o extremo esquerdo- superior, e maiores que o extremo direito-inferior. Os *labels* são delimitados por um retângulo utilizando o parâmetro *bounding box* da função *regionprops.* ^( 20 )^O retângulo intercepta cada *label* com pelo menos dois de seus vértices. Assim, se pelo menos um vértice da *bounding box* que contém o *label* estiver externo aos limites dos extremos do cérebro, isto indica que a região é externa ao cérebro e, portanto, atribui-se valor zero a todos os *pixels* de tal *label* . Com isso, eliminam-se grande parte do crânio, gordura e meninges. A figura 1 apresenta, de forma ilustrativa, o procedimento realizado para eliminação das regiões externas ao cérebro, utilizando o MDC.

**Figura 1:** Segmentação por meio do método de detecção por coordenada. À esquerda, é feita a demarcação dos pontos extremos do cérebro. No segundo quadro, é ilustrada a localização das subregiões externas e internas do cérebro. Por último, eliminam-se todas as subregiões externas ao cérebro

### Método de detecção por área

Após a aplicação da detecção por coordenadas, o algoritmo mantém subregiões na área interna ao cérebro, que não necessariamente correspondem ao tumor que se deseja segmentar. No entanto, a área do tumor cerebral resultante da detecção por coordenadas é, na maioria das vezes, maior que a média aritmética das áreas de todos os elementos espúrios que venham a permanecer. Assim, o segundo diferencial deste algoritmo é a utilização de uma propriedade bidimensional, ou seja, a área, como forma de eliminar tecidos não tumorais dentro da região do cérebro.

Antes de executar o método de detecção por área (MDA), aplicou-se a função imerode,^( 20 )^que realiza a operação morfológica de erosão com a finalidade de desconectar os elementos espúrios e reduzir suas áreas com a formação de novas subregiões com áreas menores. O MDA calcula a área de cada *label* resultante e sua média aritmética, atribuindo valor zero aos *pixels* dos elementos que possuam área menor que a média aritmética das áreas de todos os *labels* . Desta forma, elimina-se grande parte do tecido cerebral ou em sua totalidade, restando o tumor.

A figura 2 esquematiza o MDA, em que o maior quadrado em azul representa o tumor, os demais quadrados azuis representam tecidos não tumorais, e o quadrado verde representa o *label* que corresponde à média aritmética das áreas. Utilizando operações lógicas (comparações), o algoritmo verifica as regiões que possuem área menor que a área média, e as elimina, atribuindo valor lógico zero aos seus *pixels* . Desta forma, idealmente, elimina-se o número máximo de elementos indesejáveis, mantendo-se apenas o tumor.

**Figura 2:** Método da eliminação de subregiões por meio do método de detecção por área. Na primeira coluna, é feita a detecção das subregiões internas ao cérebro. Na segunda, calcula-se a média aritmética das áreas das subregiões detectadas. Na terceira, realizam-se a comparação entre o tamanho de cada subregião e a média aritmética. Na última, eliminam-se todas as subregiões com área inferior ao da média

### Pós-processamento

Utilizaram-se operadores morfológicos de dilatação ( *imdilate* )^( 20 )^para recapturar os tecidos que foram perdidos no processo de erosão. A fim de reter a maior região do tumor, a máscara binária é obtida preenchendo buracos na imagem dilatada ( *imfill* ).^( 20 )^Finalmente, a máscara binária é aplicada à imagem original, por meio de convolução espacial, para se obterem o tamanho próximo ao real dos elementos segmentados e a posterior extração dos atributos de textura.^( 15 , 23 )^

### Avaliação dos resultados

Os resultados quantitativos da segmentação automática foram calculados utilizando comparação entre o padrão-ouro e os resultados do método proposto de segmentação. As métricas comumente utilizadas na literatura^( 24 - 26 )^para análise de desempenho são a percentagem de acerto (PA), a qual é calculada em termos do número de verdadeiro-positivos (VP) e a quantidade determinada pelo padrão-ouro; e a taxa de correspondência (TC), em função do número de falso-positivos (FP). Nos trabalhos citados, a PA e a TC são definidas, respectivamente, como:

$$\mathrm{PA}=\frac{\mathrm{VP}}{\mathrm{padrão}‐\mathrm{ouro}}\times 100\%$$

$$\mathrm{TC}=\frac{\mathrm{VP}\;–\;0,5\;\times \;\mathrm{FP}}{\mathrm{padrão}‐\mathrm{ouro}}$$

Os valores da PA mais altos indicam a existência de um número maior de *pixels* verdadeiro-positivos na área segmentada. O valor de TC avalia quão distante está a segmentação do padrão-ouro. O valor ideal de TC corresponde a 1, o que indica combinação perfeita entre o padrão-ouro e a segmentação. Os valores de TC próximos a 1 indicam que a segmentação possui uma percentagem bem maior de verdadeiro-positivos com relação aos falso-positivos na segmentação.^( 16 )^

## RESULTADOS

A etapa de pré-processamento foi implementada seguindo a descrição apresentada. Ao final dela, foram obtidas as imagens tratadas com as técnicas de pré-processamento. A figura 3 mostra a saída do processo final após serem aplicados a equalização de histograma, o ajuste de intensidade para correção de contraste e a técnica de binarização. As imagens à direita são imagens binarizadas (só apresentam *pixels* com intensidade zero ou 1). São apresentados os três tipos de sequência de RM e os três tipos de cortes de fatias do encéfalo de diferentes pacientes.

**Figura 3:** Resultado das etapas de pré-processamento para pacientes distintos da base privada

A primeira, terceira e sexta colunas referem-se à imagem antes do processamento, enquanto que as demais colunas apresentam as imagens após o pré-processamento. A primeira linha mostra os resultados de imagens do tipo T1 com contraste, a segunda linha mostra os respectivos resultados para a sequência T2 e a terceira linha para a sequência T1, com contraste na sequência gradiente eco com uso de spoiller.

A etapa de segmentação teve início com a delimitação do cérebro por pontos extremos e extração das regiões externas a eles, seguida da aplicação de uma função morfológica de dilatação, e finalizando com a aplicação do algoritmo MDA. Devido à técnica de erosão, que acarreta perda de *pixels* de determinadas regiões, e ao processo de binarização, que converte a imagem em escala de cinza em apenas dois níveis de intensidade, foram utilizadas operações morfológicas de dilatação, preenchimento de buracos vazios e reconstrução, para que as regiões segmentadas fossem reconstruídas e retomadas à textura original. O procedimento morfológico foi usado nessas imagens binárias para refinar a margem e o conteúdo das imagens do tumor.

A figura 4 apresenta alguns resultados finais de total sucesso da segmentação implementada, que abrangeu exames de RM de pacientes distintos, nas ponderações T1c, T2 e FSPGR T1c nos planos axial, coronal e sagital. A segmentação de imagens na ponderação T1 com contraste para três pacientes diferentes pode ser observada na primeira linha. A segunda linha mostra os respectivos resultados das segmentações para a ponderação T2. Na terceira linha, apresentam-se os resultados de segmentação da ponderação T1 com contraste na sequência gradiente eco com uso de spoiller.

**Figura 4:** Resultado final da segmentação automática em imagens de ressonância magnética na ponderação T1, T2 e T1 com contraste na sequência gradiente eco com uso de spoiller

### Análise de desempenho comparativo da segmentação automática

As RM utilizadas no teste foram segmentadas manualmente por radiologistas. Tais resultados foram considerados padrão-ouro e utilizados para avaliar o sistema de modo eficiente. Na tabela 2 , encontram-se os percentuais de VP, FP, FN e de PA em relação ao padrão-ouro, como também os respectivos valores de TC. Em trabalhos similares, autores obtiveram PA de 86% e TC de 0,79, o que foi considerado resultado satisfatório.^( 24 - 26 )^Neste estudo, alcançou-se PA de 89,23% e TC de 0,70, ou seja, um resultado quantitativamente semelhante ao apresentado pelos autores, o que indica boa acurácia na segmentação proposta. Os melhores resultados de PA foram obtidos para as sequências T2 e FSPGR-T1c.

**Tabela 2:** Medidas de desempenho da segmentação por tipo de sequência

Sequência	PO	VP	FP	FN	PA *%*	TC
T1c	33	27	11	6	81,81	0,65
T2	48	44	19	3	91,66	0,72
FSPGR T1c	49	45	21	4	91,83	0,70
Total	130	116	51	13	89,23	0,70

PO: padrão-ouro; VP: verdadeiro-positivos; FP: falso-positivos; FN: falso-negativo; PA: percentagem de acerto; TC: taxa de correspondência.

Foram obtidos resultados também satisfatórios para a TC, embora tenha sido gerada quantidade considerável de FP. Entretanto, a ocorrência desses FP não superou a de VP, como ocorreu no estudo de Alegro et al.^( 23 )^Uma abordagem inovadora e robusta de segmentação de imagens para a extração de tumores cerebrais em IRM foi desenvolvida aplicando-se a função Kernel à máquina de vetores de suporte (K-SVM), obtendo-se PA e TC equivalentes a 87,8% e 0,85, respectivamente.^( 27 )^Para melhor comparar o desempenho do algoritmo de segmentação proposto com os encontrados na literatura, organizaram-se os resultados quantitativos de estudos semelhantes na tabela 3 .

**Tabela 3:** Medidas de desempenho de algoritmos de segmentação utilizados na literatura

Autores	Metodologia	Análise de desempenho	
		PA (%)	TC
Alegro Mde C, Amaro Junior E, Lopes Rde D. Computerized brain tumor segmentation in magnetic resonance imaging. einstein (Sao Paulo). 2012;10(2):158-63.^(23)^	SVM	94,00	-0,04
Deng W, Luo L, Lin X, Fang T, Liu D, Dan G, et al. Head and neck cancer tumor segmentation using support vector machine in dynamic contrast-enhanced MRI. Contrast Media Mol Imaging. 2017;2017:8612519.^(24)^	SVM	86,00	0,89
Singh R, Agarwal P, Bhattacharya M. MR brain tumor detection employing Laplacian Eigen maps and kernel support vector machine. In: 2016 IEEE International Conference On Bioinformatics And Biomedicine (BIBM). IEEE. 2016;827-30.^(27)^	FCM	72,80	0,43
Hsieh TM, Liu YM, Liao CC, Xiao F, Chiang IJ, Wong JM. Automatic segmentation of meningioma from non-contrasted brain MRI integrating fuzzy clustering and region growing. BMC Medical Informatics and Decision Making. 2011;11:54.^(28)^	Kernel + SVM (K-SVM)	87,80	0,85
Gao F, Lin T. Application of Computer-Aided Diagnosis Technology in Brain Tumour Detection. NeuroQuantology. 2018;16(5):725-33.^(29)^	Máquina de aprendizado	97,17	-

PA: percentagem de acerto; TC: taxa de correspondência; SVM: máquina de vetores de suporte; FCM: algortimo *fuzzy-c-mean* ; K-SVM: Kernel *support vector machine* .

## DISCUSSÃO

As técnicas de pré-processamento conseguiram eliminar grande parte do cérebro e algumas regiões do encéfalo, no entanto, ainda restaram elementos espúrios, como o osso da cabeça, os ventrículos laterais e o corpo caloso, que, em algumas situações, apresentam *pixels* com intensidades próximas ou superiores às do tecido tumoral, sendo difíceis de serem eliminados. Ainda, foi testada utilizando o filtro mediano, que é uma ferramenta muito utilizada para filtragem do ruído em RM cerebrais.^( 17 , 30 )^No entanto, não foram obtidos resultados superiores ao método desenvolvido, tendo como desvantagem o aumento do tempo de processamento do algoritmo. De fato, a operação de convolução com máscaras exige grande esforço computacional.^( 31 )^

A ponderação T1 apresenta imagens com a tonalidade do tecido tumoral muito próxima à do cérebro. Este é um problema que dificulta bastante a etapa de pré-processamento, visto que os critérios e limiares utilizados para intensificar a região tumoral acabam por ser também aplicados às demais regiões do cérebro que não são de interesse. Isto pode gerar tecidos saudáveis conectados ao tumor, mesmo após o uso de operações morfológicas, além de aumentar o número de falso-positivos após a segmentação. Os resultados do pré-processamento foram melhores nas ponderações T2 e FSPRG T1c, em consequência dessas imagens apresentarem maior contraste entre a região hiper-intensa e o restante do cérebro.

Ao analisar as figuras 3 e 4, percebe-se que o pré-processamento e a segmentação baseados nas técnicas descritas anteriormente obtiveram bons resultados com a implementação de um algoritmo simples, que utiliza funções e operações lógicas básicas. A tabela 4 apresenta a metodologia realizada por alguns autores para aprimorar a qualidade de imagens de tumores cerebrais por RM e extrair o crânio, os quais obtiveram resultados qualitativamente comparáveis aos deste estudo.

**Tabela 4:** Análise comparativa da metodologia de pré-processamento proposta com os existentes na literatura

Autores	Metodologia	Resultados
Isa IS, Sulaiman SN, Mustapha M, Karim NK. Automatic contrast enhancement of brain MR images using Average Intensity Replacement based on Adaptive Histogram Equalization (AIR-AHE). Biocybernetics and Biomedical Engineering. 2017;37(1):24-34.^(15)^	Desenvolvimento do algoritmo AIR-AHE para aprimoramento automático de contraste de RM FLAIR usando as funções *imadjust* e *stretchlim*	Apresentou bons resultados em conjunto com outros algoritmos de equalização de histograma
Isselmou AE, Zhang S, Xu G. A novel approach for brain tumor detection using MRI Images. J Biomedical Sci Eng. 2016;9(10):44-52.^(17)^	Uso do filtro de mediana e de passa-alta, e equalização de histograma para melhorar a qualidade das imagens	Permitiu melhorar a qualidade das imagens, obtendo excelentes resultados na segmentação do tumor
Sujan M, Alam N, Noman SA, Islam MJ. A Segmentation based Automated System for Brain Tumor Detection. IJCA. 2016;153(10):41-9.^(32)^	Extração do cérebro em imagens FLAIR usando as operações morfológicas do MATLAB^®^, como binarização, erosão, dilatação e elementos estruturantes	O método contribuiu para melhores resultados no aprimoramento de imagens, apresentando incremento na acurácia comparado a outros algoritmos da literatura
Roy S, Maji P. A simple skull stripping algorithm for brain MRI. In: Eighth International Conference On Advances In Pattern Recognition (ICAPR) [Internet]. Kolkata: (IN); 2015 [cited 2019 Aug 20]. Available from: https://ieeexplore.ieee.org/abstract/document/7050671/^(33)^	Desenvolvimento de um método de extração do crânio (S3) para ponderação T1 baseada nas características da anatomia do cérebro e na intensidade da imagem	O desempenho do algoritmo é comparado a BET e BSE, obtendo resultados satisfatórios
khandelwal P, Kaur G. Comparative study of different image enhancement technique. IJECT. 2016;7(2):116-21.^(34)^	Comparação de técnicas de aprimoramento de contraste (subtração, ajuste de contraste, erosão, correção *gamma* , inversão e limiarização)	A técnica da erosão produziu o melhor resultado
Kaur R, Chawla M, Khiva NK, Ansari MD. Comparative Analysis of Contrast Enhancement Techniques for Medical Images. Pertanika J Sci Technol. 2018;26(3):965-78.^(35)^	Comparação de técnicas de aprimoramento de contraste (operação de vizinhança, filtro médio, *imadjust* e função sigmoide)	A função sigmóide e a operação de vizinhança produziram o melhor resultado
Shattuck DW, Sandor-Leahy SR, Schaper KA, Rottenberg DA, Leahy RM. Magnetic resonance image tissue classification using a partial volume model. Neuroimage. 2001;13(5):856-76.^(36)^	Desenvolvimento de um método de extração do crânio (BSE) em imagens de ponderação T1 que utiliza um detector de borda e uma série de operações morfológicas	Método robusto que contribuiu para segmentação da GM e WM do cérebro, respectivamente
Smith SM. Fast robust automated brain extraction. Hum Brain Mapp. 2002;17(3):143-55. Review.^(37)^	Desenvolvimento de um método de extração do crânio (BET) em imagens T1	Método robusto e preciso aplicado a uma variedade de sequências RM
Roura E, Oliver A, Cabezas M, Vilanova JC, Rovira A, Ramió-Torrentà L, et al. MARGA: multispectral adaptive region growing algorithm for brain extraction on axial MRI. Comput Methods Programs Biomed. 2014 Feb;113(2):655-73.^(38)^	Desenvolvimento de um método de extração do crânio (MARGA) em vistas axiais, baseado no crescimento de região	O MARGA apresentou resultados superiores quando comparado com as abordagens BET e BSE
Somasundaram K, Mercina JH, Magesh Kalaiselvi ST. Brain Portion Extraction Scheme using Region Growing and Morphological Operation from MRI of Human Head Scans. IJCSE. 2018;6(4):298-302.^(39)^	Desenvolvimento de um método de extração do crânio baseado no crescimento de região e operações morfológicas (erosão, dilatação e preenchimento)	Os resultados do método são melhores do que os métodos existentes (BET e BSE)
Kalavathi P, Prasath VB. Methods on Skull Stripping of MRI Head Scan Images—a Review. J Digit Imaging. 2016;29(3):365-79. Review.^(40)^	Desenvolvimento de um método de extração do cérebro de RM em T1 baseado no filtro médio e em operações morfológicas	O método deu resultados comparáveis com os métodos BET e BSE e mostrou o uso do filtro médio não impactou melhoras na segmentação

AIR-AHE: *Average Intensity Replacement based on Adaptive Histogram Equalization* ; RM: imagem de ressonância magnética; FLAIR: *fluid attenuation inversion recovery* ; MATLAB^®^: *MATrix LABoratory* ^®^; BET: *brain extraction tool* ; BSE: *brain surface extractor* ; GM: substância cinzenta; WM: substância branca; MARGA: *multispectral adaptive region growing algorithm* .

O algoritmo MDC é capaz de marcar os extremos do cérebro em qualquer tipo de corte (axial, coronal ou sagital) do encéfalo. Isso torna o sistema mais preciso, visto que a forma geométrica do cérebro varia nos três tipos de corte. Ainda, ele funciona também quando ocorre assimetria na localização da cabeça dentro da imagem, seja horizontal ou verticalmente, o que é de comum ocorrência.^( 30 , 41 )^Ademais, abrange diversas possibilidades para extração das regiões externas ao cérebro, aplicando-se a imagens com hipersinal em distintas ponderações, visto que independe de características qualitativas, como entropia e textura. Ao contrário, ele é baseado na localização em que o encéfalo se encontra dentro da imagem, em sua anatomia e intensidade dos *pixels* circundantes, semelhante ao que foi proposto no método S3.^( 33 )^

O MDC apresentou resultados satisfatórios quanto à sua funcionalidade e à sua abrangência, podendo ser comparado, qualitativamente, com os algoritmos apresentados na tabela 4 , que têm bom desempenho na extração do crânio. Nos casos em que a área externa ao cérebro não é completamente eliminada, a operação morfológica de dilatação é capaz de subdividir tais “sobras” em partes menores, que podem ser eliminadas com a aplicação do MDA. Assim, o MDA executa a tarefa de complemento à segmentação por detecção de coordenadas. Ele está diretamente ligado à quantidade de FP existentes na pós-segmentação, visto que quanto mais tecidos não tumorais forem eliminados, menor a quantidade de FP e melhor o desempenho da segmentação.

No entanto, o MDA é falho em situações que existem regiões pós-processadas com área maior que o tumor, pois há o risco de a área do tumor ser inferior à área média das subregiões existentes e causar sua eliminação, reduzindo a quantidade de VP; ou produzir imagens com tumor e não tumor, aumentando o número de FP. Isso pode acontecer em fatias em que os tecidos normais possuem intensidades de *pixel* semelhantes às intensidades dos *pixels* do tumor, e nas quais a diferenciação dessas regiões é dificultada com as técnicas de pré-processamento adotadas. Além dessas limitações, o MDC e o MDA foram desenvolvidos para segmentar apenas RM com tumor em hipersinal.

A sequência T1c apresentou valores de PA e TC mais baixos comparados às demais ponderações, por apresentaram imagens em que a região cerebral é composta por *pixels* com intensidades muito semelhantes ao do tumor. Com isso, foi gerado um número elevado de FP conectados, que, em alguns casos, possuíam área muito superior à do tumor; desta forma, o tumor foi eliminado, e a imagem segmentada não apresentou tecido tumoral, gerando os FN.

Os casos falhos utilizando a ponderação T2 devem-se à presença de ventrículos em algumas fatias de corte coronal e dos olhos e nariz, em fatia axial, que apresenta *pixels* com intensidade muito alta, quase próxima a 1, o que acarretou elevado número de FP, além do tumor não ter sido detectado em três fatias FN. Este último problema também acarretou falhas em fatias no plano axial da ponderação FSPGR T1c, explicando seu elevado número de FP.

Embora tenha sido gerada uma quantidade considerável de FP, foram obtidos resultados satisfatórios para PA e TC, alcançando-se média de 89,23% de acertos entre as áreas segmentadas automaticamente e o padrão-ouro, o que indica boa acurácia na segmentação proposta.

## CONCLUSÃO

Os resultados obtidos mostraram que o sistema proposto foi capaz de localizar e delimitar a região tumoral sem qualquer interação com o usuário, utilizando método inovador de segmentação automática, simples e fácil implementação.
